# NIR responsive tumor vaccine in situ for photothermal ablation and chemotherapy to trigger robust antitumor immune responses

**DOI:** 10.1186/s12951-021-00880-x

**Published:** 2021-05-17

**Authors:** Lirong Zhang, Jingjing Zhang, Lixia Xu, Zijian Zhuang, Jingjin Liu, Suwan Liu, Yunchao Wu, Aihua Gong, Miaomiao Zhang, Fengyi Du

**Affiliations:** 1 Department of Radiology, Affiliated Hospital of Jiangsu University, Zhenjiang, People’s Republic of China; 2 School of Medicine, Jiangsu University, Zhenjiang, Jiangsu People’s Republic of China; 3 The Third People’s Hospital of Changzhou, Changzhou, People’s Republic of China

**Keywords:** Polydopamine nanoparticle, Hyaluronic acid, Photothermal therapy, Immune therapy

## Abstract

**Background:**

Therapeutic tumor vaccine (TTV) that induces tumor-specific immunity has enormous potentials in tumor treatment, but high heterogeneity and poor immunogenicity of tumor seriously impair its clinical efficacy. Herein, a novel NIR responsive tumor vaccine in situ (HA-PDA@IQ/DOX HG) was prepared by integrating hyaluronic acid functionalized polydopamine nanoparticles (HA-PDA NPs) with immune adjuvants (Imiquimod, IQ) and doxorubicin (DOX) into thermal-sensitive hydrogel.

**Results:**

HA-PDA@IQ NPs with high photothermal conversion efficiency (41.2%) and *T*_1_-relaxation efficiency were using HA as stabilizer by the one-pot oxidative polymerization. Then, HA-PDA@IQ loaded DOX via π-π stacking and mixed with thermal-sensitive hydrogel to form the HA-PDA@IQ/DOX HG. The hydrogel-confined delivery mode endowed HA-PDA@IQ/DOX NPs with multiple photothermal ablation performance once injection upon NIR irradiation due to the prolonged retention in tumor site. More importantly, this mode enabled HA-PDA@IQ/DOX NPs to promote the DC maturation, memory T cells in lymphatic node as well as cytotoxic T lymphocytes in spleen.

**Conclusion:**

Taken together, the HA-PDA@IQ/DOX HG could be served as a theranostic tumor vaccine for complete photothermal ablation to trigger robust antitumor immune responses.

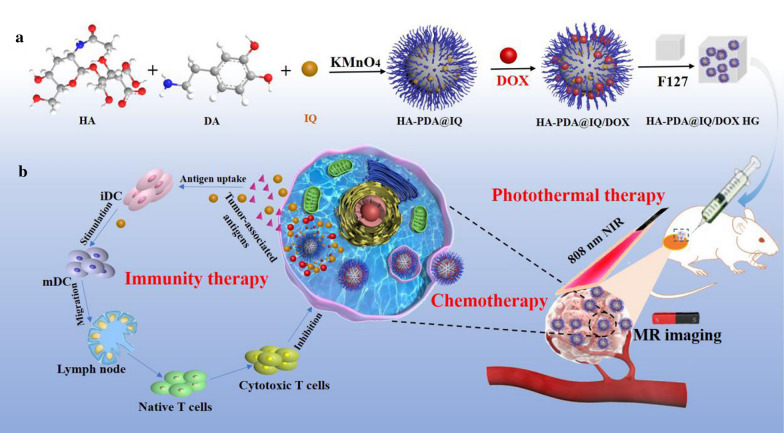

## Introduction

Therapeutic cancer vaccine (TCV) that aims to stimulate tumor antigen-specific immunological responses is emerging as promising technology in cancer immunotherapy in recent years [1–3]. In particular, substantial tumor antigen-based tumor vaccines have been deeply explored in basic research and clinic trials, resulting in the approval of the prostatic acid phosphatase-based peptide vaccination by the U.S. Food and Drug Administration [4–6]. Despite great progress, the therapeutic effect of TCV is impaired due to the complex physiological characteristics of tumor, including high heterogeneity with various somatic mutations and corresponding neoantigens among patients [7–9]. Thus, it is significant to overcome these hurdles to eliminate and inhibit cancer.

Personalized TCV in situ represents a promising strategy to overcome tumor heterogeneity by inducing autologous whole tumor antigens release [10–12]. Up to now, a variety of immuno-stimulatory treatments for development of TCV, such as oncolytic viruses [13, 14], radiotherapy [15, 16], chemotherapy [17] and phototherapy [18, 19], have been reported to generate tumor-associated antigens in situ by inducing immunogenic cell death (ICD) [20–22]. Among these treatments, near infrared (NIR) laser mediated photothermal ablation (PTA) has been regarded as a powerful technique due to its high selectivity and minimal side effects [23, 24]. Its therapeutic effects required both the photosensitizer accumulation at the tumor site and localized NIR laser exposure, leading to the heating temperature up to 50 ℃ to realize tumor coagulative necrosis [25, 26]. In addition, numerous studies have demonstrated that photothermal therapy could cause immunogenic death of tumor cells (ICB) and induce tumor-derived antigens release. However, the uptake and recognition of these tumor-derived antigens by antigen presenting cells was severely impaired due to tumor immunosuppressive microenvironment. Therefore, the ineffectivity of PPT-triggered antitumor immune response suggested that additional therapeutic interventions may be needed to develop the combination therapy against for recurrence and metastasis of tumor [27, 28].

Mounting evidences have demonstrated that simulation of dendritic cells (DCs) with immunoadjuvants could promote the antigens presentation and immune response to relieve immunosuppression [29, 30]. Mature DCs capture antigens derived from cancer and assembled with the major histocompatibility complex (MHC) to trigger robust CD8^+^ cytotoxic T lymphocyte (CTL) response [31, 32]. For instance, imiquimod (R837) as a potent TLR7 agonist has been integrated into distinct TCV nanosystem to collaborate with radiotherapy [33–35], photothermal therapy [36], photodynamic therapy (PDT) [37, 38], and chemotherapy [39]. However, the actual therapeutic effects of these TCV were impaired due to their complicated manufacture process and unsatisfactory performance.

In order to address above limitations, NIR responsive cancer vaccine in situ (HA-PDA@IQ/DOX HG) was fabricated to stimulate strong antitumor immunity. Briefly, hyaluronic acid (HA) and imiquimod (IQ) were integrated into polydopamine nanoparticles (PDA NPs) using potassium permanganate mediated-one step polymerization. A certain amount of DOX were adsorbed on the surface of PDA NPs through the interaction of π–π accumulation and electrostatic attraction. In our expectation, HA could be used to improve the biocompatibility and stability of polydopamine nanoparticles, and the released DOX under acidic conditions further destroy residual tumor cells after PTA. Moreover, the immune adjuvant IQ synergized with PTA-triggered tumor-derived antigens to promote the maturation of dendritic cells in the body and enhance anti-tumor immunity response. KMnO_4_ could not only catalyze the formation of PDA nanoparticles, but also introduced the Mn-based MR imaging capacity to realize the integration of tumor diagnosis and treatment. In this study, the chemical and physical properties of HA-PDA@IQ/DOX NPs were characterized and its photothermal conversion efficiency was calculated. The ability of HA-PDA@IQ/DOX NPs to induce dendritic cells (DCs) maturation was studied using bone morrow derived dendritic cells (BMDCs) in vitro. Furthermore, thermosensitive hydrogel loaded HA-PDA@IQ/DOX NPs was injected in situ into tumor site to investigate the influence on tumor growth and immune system.

## Results

### Preparation and characterization of HA-PDA@IQ/DOX NPs

**Scheme 1.**
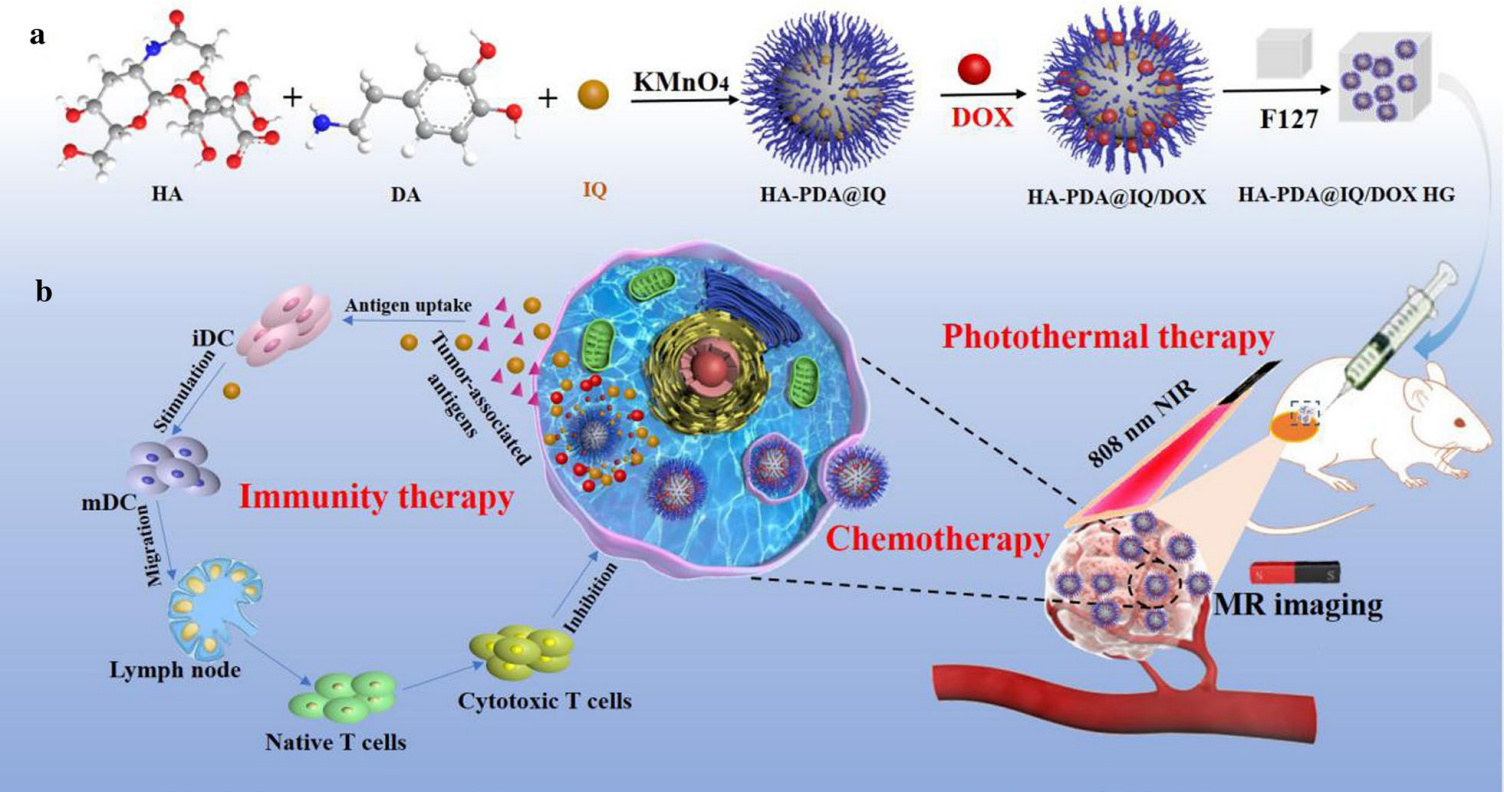
Schematic illustration of preparation process of HA-PDA@IQ/DOX HG (**a**) and in vivo anti-tumor applications (**b**)

**Fig. 1:**
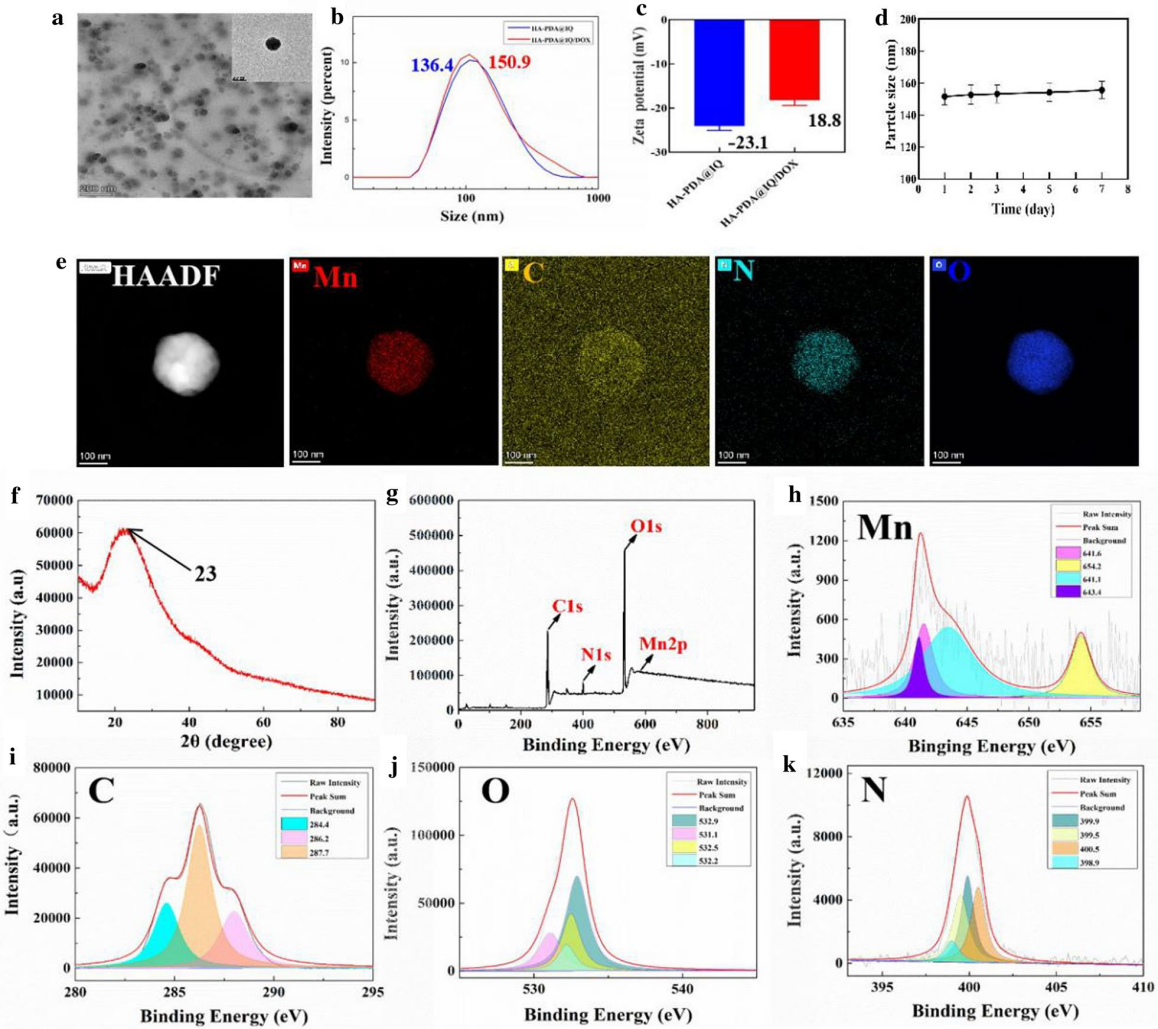
Characterizations of HA-PDA@IQ/DOX NPs. **a** Typical TEM images, Scale bar: 200 nm. **b** Particle size distribution, **c** Surface zeta potential, **d** In vitro stability, **e** FETEM images and elemental mapping images (Mn, C, N, and O), Scale bar:100 nm, **f** XRD spectra, **g** XPS spectra of HA-PDA@IQ/DOX NPs. **h** Peak-fitting spectra for Mn2p, **i** C1s, **j** O1s, **k** N1s of HA-PDA@IQ/DOX NPs

As shown in Scheme 1, HA-PDA@IQ NPs were synthesized by the one-pot self-polymerization of dopamine initiated by KMnO_4_ in the presence of stabilizer (HA) and immune adjuvant (IQ). Then, the chemotherapy drug (DOX) was loaded via π-π stacking to form HA-PDA@IQ/DOX NPs. As shown in Fig. 1a, the TEM result showed that the prepared HA-PDA@IQ/DOX NPs were spherical, uniformly distributed without obvious aggregation. The average sizes and zeta potentials of HA-PDA@IQ NPs and HA-PDA@IQ/DOX NPs were 136.4 nm and 150.9 nm, − 23.1 mV and − 18.8 mV, respectively (Fig. 1b, c). The changes in size and zeta potential were attributed to DOX loading. Furthermore, HA-PDA@IQ/DOX NPs showed great size stability under PBS during one week (Fig. 1d). In addition, elemental mapping images displayed that Mn element has been successfully integrated into HA-PDA@IQ/DOX NPs during oxidative polymerization (Fig. 1e). XRD spectrum indicated that there was broad diffraction peak at 23°, which represented non crystalline phase structure in HA-PDA@IQ/DOX NPs (Fig. 1f**)**. As shown in Fig. 1g, the chemical structure and composition of HA-PDA@IQ/DOX NPs were analyzed by X-ray photoelectron spectroscopy (XPS). There were four peaks at 641.48 eV, 532.59 eV, 399.88 eV and 296.29 eV, corresponding to the four elements of manganese, oxygen, nitrogen and carbon, which proved the element composition of HA-PDA@IQ/DOX NPs. As shown in Fig. 1h, there were two typical binding-energy peaks at 654.2 eV and 641.1 eV, corresponding to Mn (IV)_2p1/2_ and Mn (IV)_2p3/2_, respectively, which further confirmed the presence of Mn element in HA-PDA@IQ/DOX NPs. The C1s spectrum showed three main peaks, 287.7 eV, 286.2 eV, and 284.4 eV (Fig. 1i), corresponding to C=0, C–N and C–C, respectively. The O1 spectrum had two obvious peaks at 532.98 eV and 531.1 eV, which were attributed to C =O and C-O (Fig. 1j**)**. The N1s spectrum showed four peaks, 399.9 eV, 399.5 eV, 398.9 eV and 400.5 eV, which indicated the presence of N atoms (Fig. 1k**)**. These hydrophilic chemical groups endowed the HA-PDA@IQ/DOX NPs with superior dispersibility and stability in aqueous solution, which were essential for biomedical applications.

### Photothermal properties of HA-PDA@IQ/DOX NPs

**Fig. 2:**
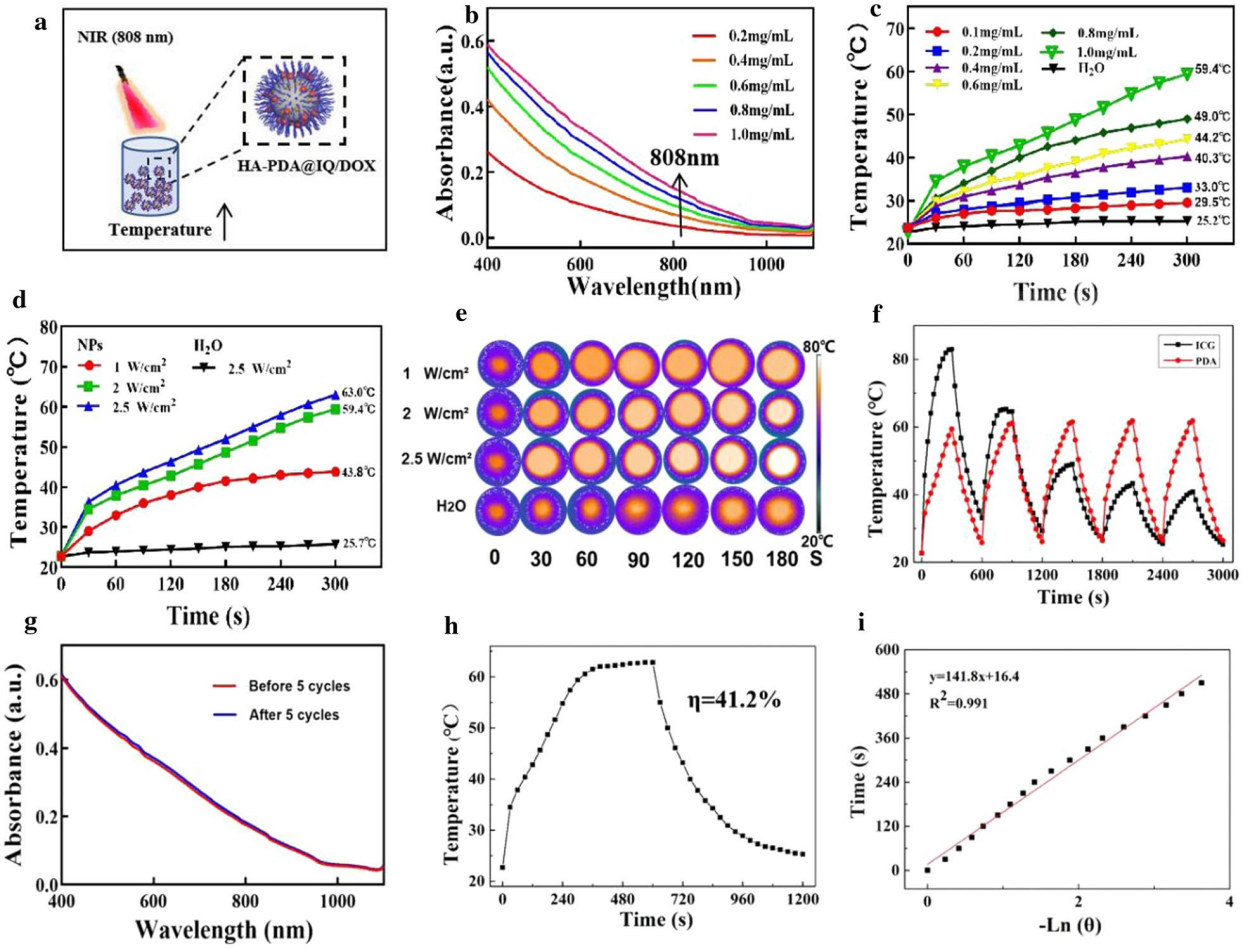
In vitro photothermal performance of HA-PDA@IQ/DOX NPs. **a** Diagram of the photothermal effect of HA-PDA@IQ/DOX NPs. **b** Vis–NIR absorbance spectra of HA-PDA@IQ/DOX. **c** Photothermal heating curves of HA-PDA@IQ/DOX at different concentrations and **d** under varied power densities. **e** Thermal imaging of HA-PDA@IQ/DOX NPs solution under different laser power density. **f** Periodical temperature changes of the HA-PDA@IQ/DOX NPs solution upon laser irradiation for five laser on/off cycles and **g** following Vis–NIR absorbance spectra. **h** Photothermal response of HA-PDA@IQ/DOX NPs solution treated with an NIR laser (808 nm, 2 W/cm^2^) for 600 s and left to cool down. **i** Linear time data versus − ln θ obtained from the cooling stage

Photothermal conversion performance of HA-PDA@IQ/DOX NPs was investigated upon 808 nm laser irradiation (Fig. 2a). Firstly, the UV–Vis spectra of HA-PDA@IQ/DOX NPs showed a wide absorption band extending to the infrared region, where the absorption intensity increased with the increase of concentration (0.2–1.0 mg/mL) in Fig. 2b. Next, we studied the temperature changes of HA-PDA@IQ/DOX NPs solution with different concentrations under laser irradiation of 2 W/cm^2^, as shown in Fig. 2c. Generally, the temperature of each group from 0.1 to 1.0 mg/mL quickly increased with the increase of HA-PDA@IQ/DOX NPs concentration. By contrast, there was no obvious change in temperature in de-ionized water group. Notably, the temperature of HA-PDA@IQ/DOX NPs group even reached 59.4 °C within 5 min under 808 nm laser irradiation when the concentration was 1 mg/mL. In addition, we tested the temperature changes of HA-PDA@IQ/DOX NPs (1 mg/mL) group at different power densities from 1 to 2.5 W/cm^2^ (Fig. 2d), and recorded the heat map using an infrared camera (Fig. 2e). As we expected, the temperature exhibited power density-dependent manner, even exceeded 60 °C under the power density of 2.5 W/cm^2^. Compared with commercial photosensitizer ICG, photothermal stability of HA-PDA@IQ/DOX NPs (200 μL, 1.0 mg/mL) was studied during the five cycles of on/off laser. As shown in Fig. 2f, the temperature of the ICG solution gradually decreased after five rounds of repeated laser irradiation due to inherent photoquenching. However, the temperature of the HA-PDA@IQ/DOX NPs solution remained stable during five irradiation cycles, indicating that HA-PDA@IQ/DOX NPs have a highly stable photothermal effect. Besides, there was no significant change in the Vis–NIR absorbance of HA-PDA@IQ/DOX NPs before and after five rounds of repeated laser irradiation (Fig. 2g). According to the calculation formula in Fig. 2h and i, the photothermal conversion efficiency (η) of HA-PDA@IQ NPs was 41.2%, which was obviously much higher than those of previously reported PTT agents [40]. The high η of HA-PDA@IQ/DOX NPs could be attributed to its strong absorption at the near-infrared (NIR) wavelength of 808 nm and the increase in electron transport efficiency induced by manganese element.

### Dox loading and release

**Fig. 3:**
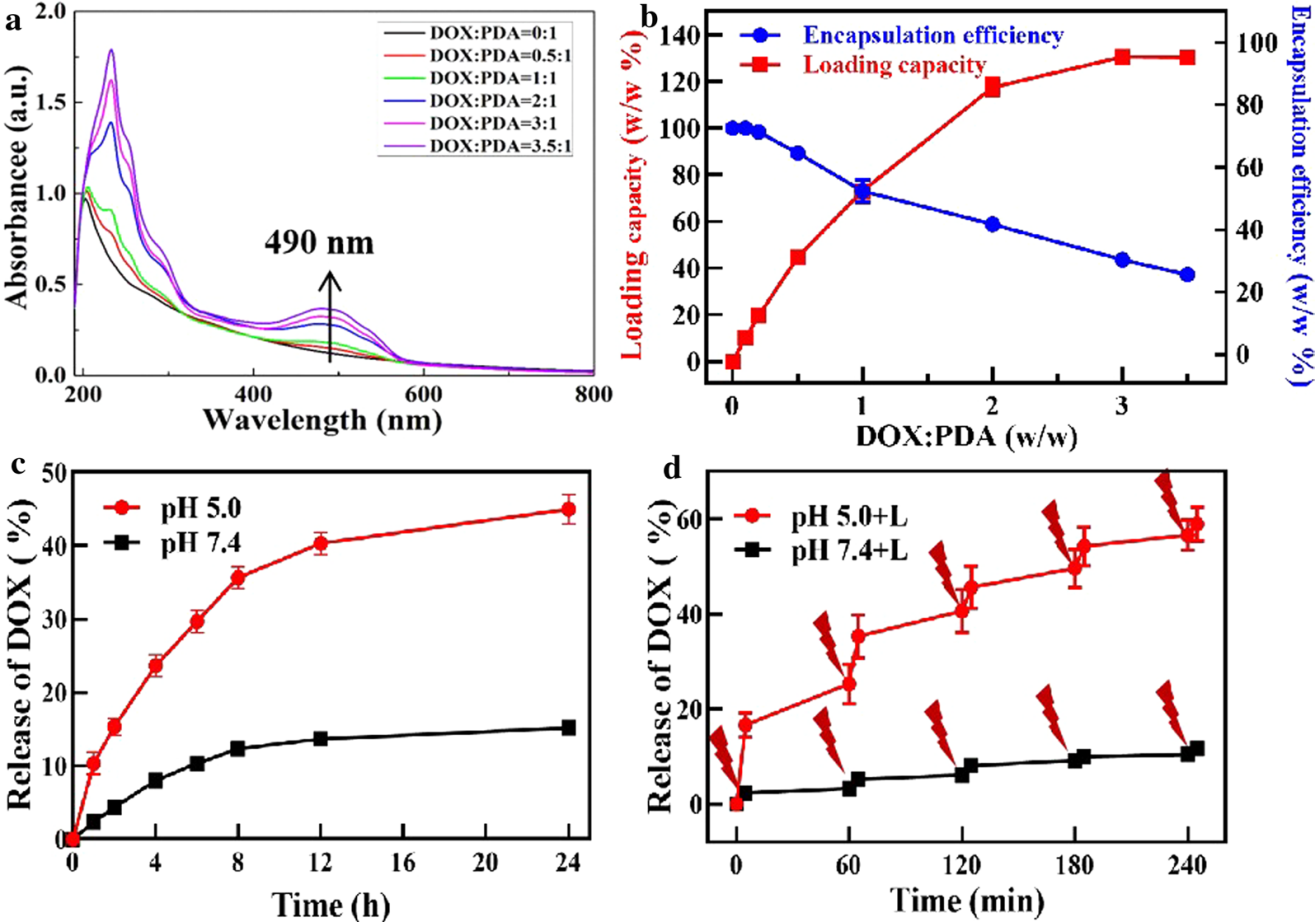
The loading and release profiles of DOX. **a** UV–Vis-NIR spectra of HA-PDA@IQ/DOX NPs with different feeding ratios of DOX to HA-PDA@IQ. **b** Quantification of DOX loading from HA-PDA@IQ/DOX NPs at different ratios of DOX: HA-PDA@IQ. **c** DOX release from HA-PDA@IQ/DOX NPs at the different pH values. **d** NIR-triggered release of DOX from HA-PDA@IQ/DOX NPs

It is well known that aromatic molecules can be efficiently loaded on nanoparticles with delocalized π electrons through π-π stacking and hydrophobic interaction. Since PDA contained a delocalized π electronic structure, we envisioned that HA-PDA@IQ can also be used as a nano-drug delivery carrier to load the doxorubicin (common chemotherapy drug, DOX). In our experiment, HA-PDA@IQ and DOX were mixed in different ratios and stirred overnight at room temperature. After washing, the obtained HA-PDA@IQ/DOX NPs were detect by UV–Vis-NIR spectroscopy (Fig. 3a). Compared with pure HA-PDA@IQ NPs, the HA-PDA@IQ/DOX NPs has a characteristic absorption peak at 490 nm, indicating that DOX was successfully loaded. As the weight ratio of DOX: PDA increased, the drug loading on the nanoparticles also increased (Fig. 3b). The maximum loading reached about 130% (DOX: PDA, w/w), which seemed to be much higher than traditional polymer-based NDDS.

The drug release behavior of DOX in HA-PDA@IQ/DOX NPs.The supernatant was collected by dissolving HA-PDA@IQ/DOX in PBS with different pH values (5.0 and 7.4) with gentle agitation (Fig. 3c). The amount of released DOX was analyzed by UV–Vis-NIR spectroscopy. After incubation of 24 h, the released DOX at pH 5.0 was about 45%, while the released DOX at pH 7.4 was only 15.2%. Therefore, DOX release seems to be more pronounced at a lower pH (5.0) compared with physiological pH (7.4). This acid-triggered release was attributed to the protonation of the amino group in the DOX molecule under low pH. Then, we wondered whether the NIR-induced heating could promote the release of DOX. The HA-PDA@IQ/DOX NPs in pH 5.0 and 7.4 PBS were irradiated by 808 nm laser (2 W/cm^2^, 5 min) at different time points. As shown in Fig. 3d, the cumulative release of DOX in HA-PDA@IQ/DOX NPs was measured by UV–Vis-NIR spectroscopy. Compared with no laser irradiation treatment, released DOX exhibited periodical fluctuation along laser on/off irradiation, demonstrating that irradiation could significantly enhanced the release of DOX from HA-PDA@IQ/DOX NPs. In view of these features, acidic environment of tumor tissue and HA-PDA@IQ/DOX NPs-mediated photothermal effect were conducive to the controllable release of DOX.

### Biocompatibility and PTA effect of HA-PDA@IQ NPs in vitro

**Fig. 4:**
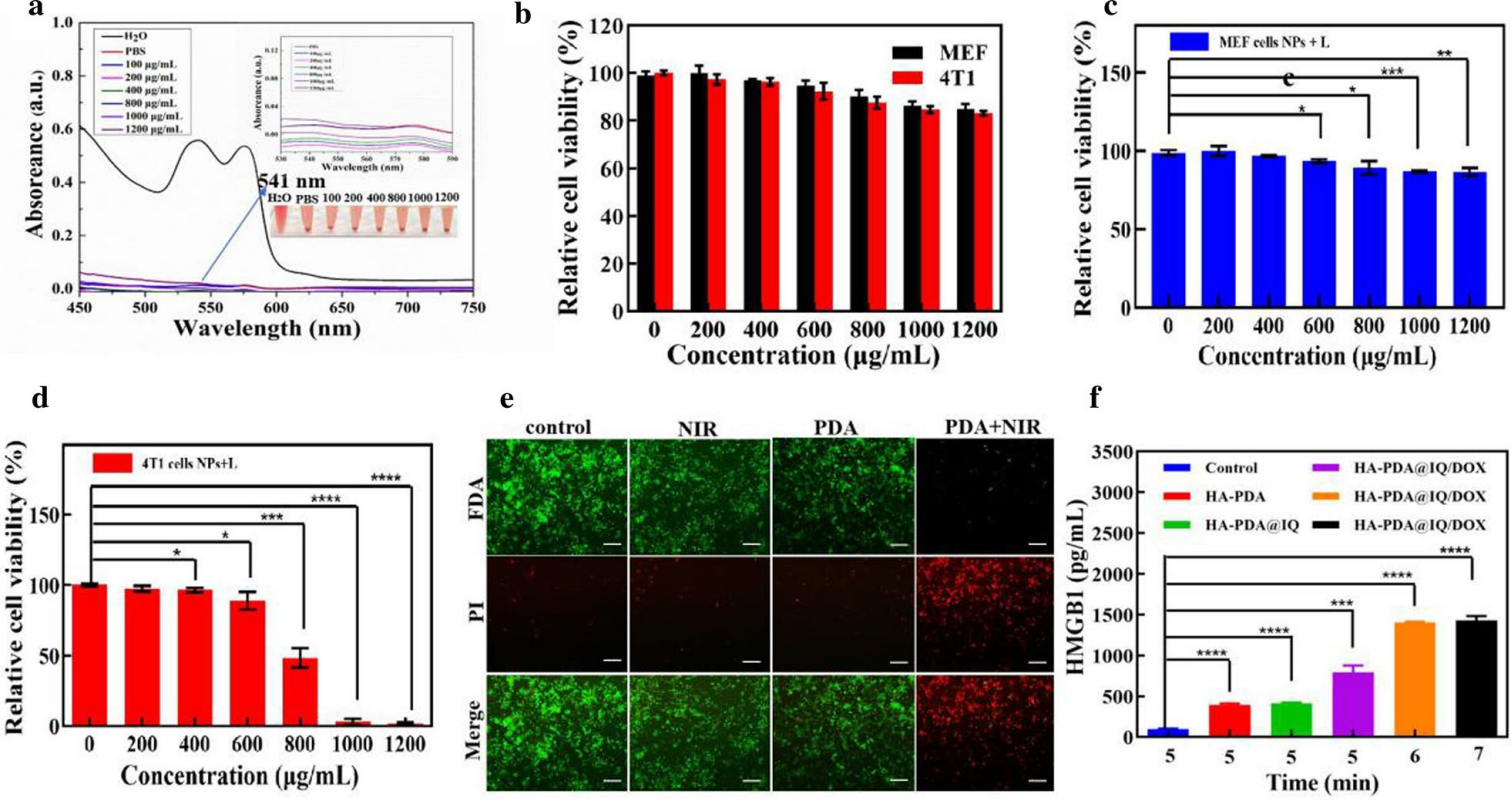
**a** Hemolytical activity of the HA-PDA@IQ at different concentrations. PBS and water were used as negative and positive control, respectively. **b** Cell viability of 4T1 cells and MEF cells co-cultured with different concentrations of HA-PDA@IQ NPs for 48 h. **c** Relative viability of 4T1 cells and **d** MEF cells after HA-PDA@IQ NPs upon laser irradiation (2 W/cm^2^, 5 min) at various concentrations. **e** Fluorescent images of 4T1 cells in different treatment groups by live (green) and dead (red) cell staining. Scale bar:100 μm. **f** The release of HMGB1 in different treatment groups. *p* values were calculated by the Student’s t test. Mean ± SD (n = 4) of three independent experiments, *p < 0.05, **p < 0.01, ***p < 0.001, ****p < 0.001

In order to eliminate the interference of DOX, HA-PDA@IQ NPs without DOX was used in the following experiments. To evaluate the blood biocompatibility, the hemolysis test was used to evaluate the blood compatibility of HA-PDA@IQ NPs. As shown in Fig. 4a, compared with the deionized water group, the absorbance at 541 nm did not exceed 0.04 even at 1200 μg/mL. Furthermore,two types of cells, including MEF cells (normal mouse embryonic fibroblast) and 4T1 cells (mouse breast cancer cells) were also performed to evaluate the biocompatibility.Both MEF cells and 4T1 cells were cultured with different concentrations of HA-PDA@IQ NPs (0, 200, 400, 600, 800, 1000, 1200 μg/mL) for 48 h, respectively. As shown in Fig. 4b, HA-PDA@IQ NPs showed negligible cytotoxicity to both MEF cells and 4T1 cells. Even at a high concentration of 1200 μg/mL, both these cell survival rate exceeded 80%. These above results indicated that the prepared HA-PDA@IQ NPs has good biocompatibility.

After that, we evaluated the effect of HA-PDA@IQ NPs mediated PTA on tumor growth in vitro. The tumor cells 4T1 and normal cell MEF were co-cultured with different concentrations of HA-PDA@IQ NPs and irradiated with 808 nm laser for 5 min (2 W/cm^2^). It can be seen from Fig. 4c and d that the cell viability of 4T1 cells was lower than 50% at 800 μg/mL, while the cell viability of MEF cells was even higher than 80% at same concentration. The findings proved that PTA significantly inhibited the tumor cell viability, but has little effect on normal cells. The possible reason was that normal cells are more resistant to temperature than cancer cells. In addition, live/dead cell assay was conducted to verify the effect of PTA on tumor cells. It can be seen from Fig. 4e that there was almost no cell death in control, NIR and HA-PDA@IQ NPs treatment. In contrast, HA-PDA@IQ NPs + NIR caused mass cell death, indicating that HA-PDA@IQ NPs had promising potential in PTA of tumor. Furthermore, high mobility group box 1 (HMGB1) as apoptotic biomarker from 4T1 cells was measured using ELISA assay in Fig. 4f. After 5 min of irradiation, the HMGB1 in HA-PDA@IQ/DOX group had the highest release of 0.799 μg/mL. With the increase of the irradiation time from 6 to 7 min, the release of HMGB1 in HA-PDA@IQ/DOX group showed an increasing trend and was stable around 1.40 μg/mL.

### The effect of HA-PDA@IQ NPs on DC maturation.

**Fig. 5:**
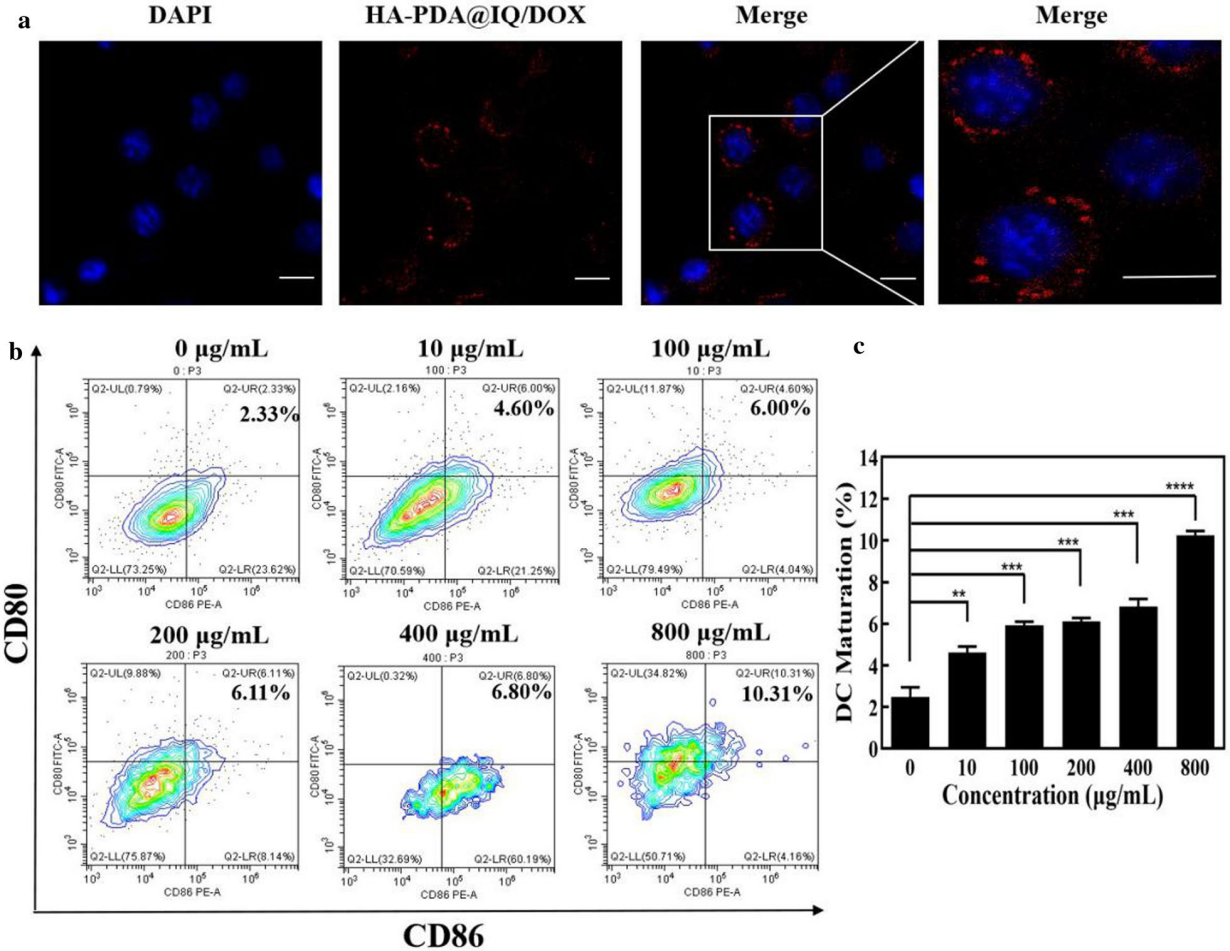
**a** LSCM images of 4T1 cells incubated with 100 μg/mL HA-PDA@IQ/DOX NPs. Scale bar: 10 μm. **b** Flow cytometry analysis of CD80 and CD86 on the BMDCs surface after cocultured with HA-PDA@IQ NPs at different concentrations. **c** Statistic data according to **b**. *p* values were calculated by the Student’s t test. Mean ± SD (n = 4) of three independent experiments, *p < 0.05, **p < 0.01, ***p < 0.001, ****p < 0.001

Firstly, the intracellular localization and real-time cell uptake of HA-PDA@IQ/DOX NPs were studied via fluorescence microscopy after incubation 4T1 cells with HA-PDA@IQ/DOX NPs for 24 h. As shown in Fig. 5a, red fluorescence could be observed in the cytoplasm, but no signal in nucleus. These findings showed that HA-PDA@IQ/DOX NPs could be phagocytosed into the cytoplasm and distributed around nucleus.

During the antitumor immune response, DC as a type of antigen presenting cell (APC) plays an essential role in capturing the neoantigen and activating cytotoxicity T lymphocyte. Therefore, immature bone marrow-derived dendritic cells (BMDC) were used to evaluate the effect of HA-PDA@IQ NPs on DC maturation. Flow cytometry analysis was used to study the expression of CD80 and CD86 on the surface of BMDCs, which represented the level of DC maturation. Figure 5b and c showed the expression of CD80 and CD86 on the surface of BMDCs increased with the increase of HA-PDA@IQ NPs concentration. Compared with PBS treatment (only 2.33%), CD80 and CD86 expression in HA-PDA@IQ NPs treatment was up to 10.31%. Based on these findings, we have reason to believe that resultant HA-PDA@IQ NPs have a powerful effect on triggering DC maturation, which may be beneficial to anti-tumor immune activation.

### MRI performance of HA-PDA@IQ/DOX NPs in vitro and in vivo

**Fig. 6:**
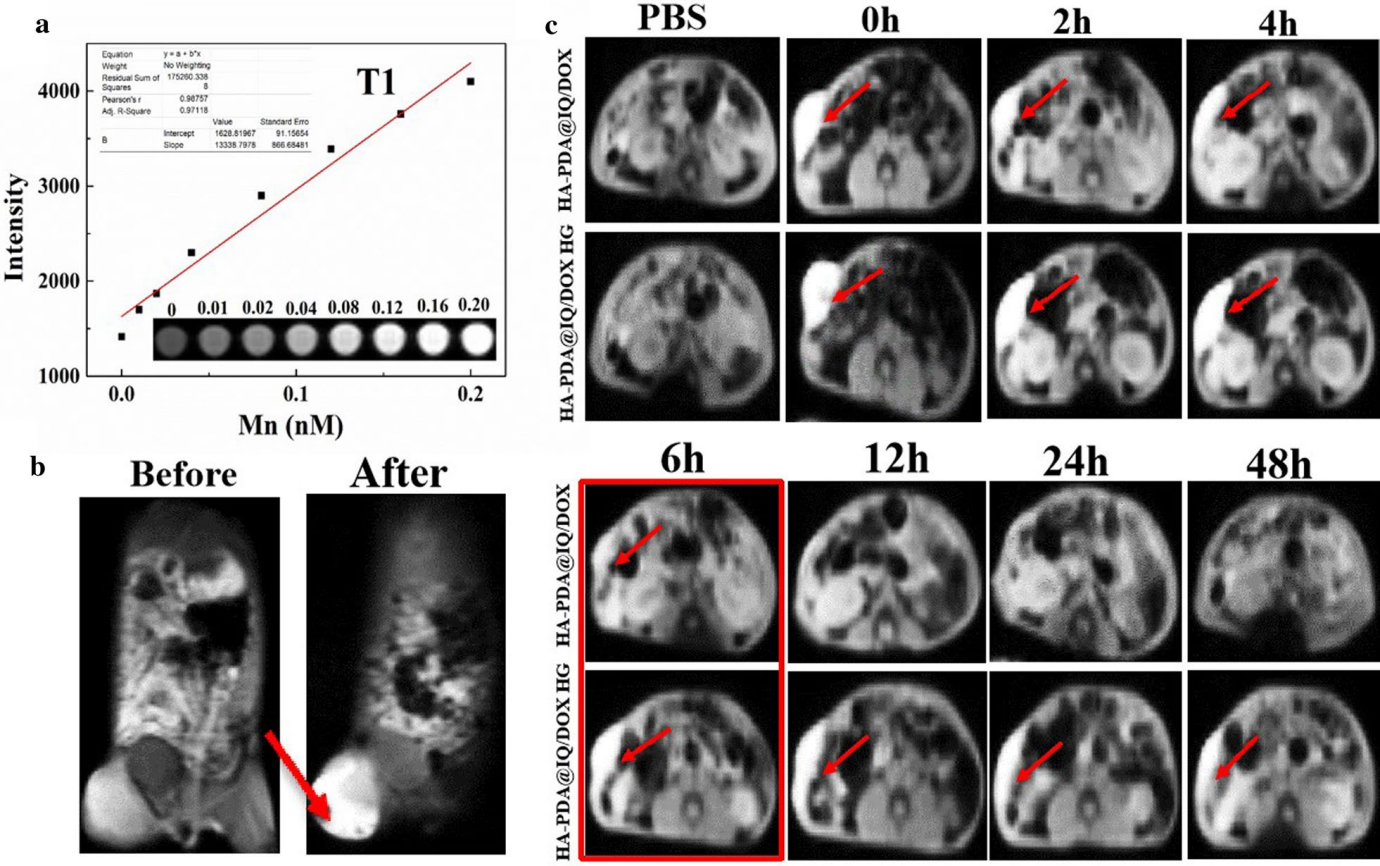
**a** *T*_1_-weighted MR images of HA-PDA@IQ/DOX containing various Mn concentration. **b** *T*_1_-weighted MR images of HA-PDA@IQ/DOX HG at the tumor site. **c** *T*_1_-weighted images of HA-PDA@IQ/DOX and HA-PDA@IQ/DOX HG retention in the tumor site within 48 h

**Fig. 7:**
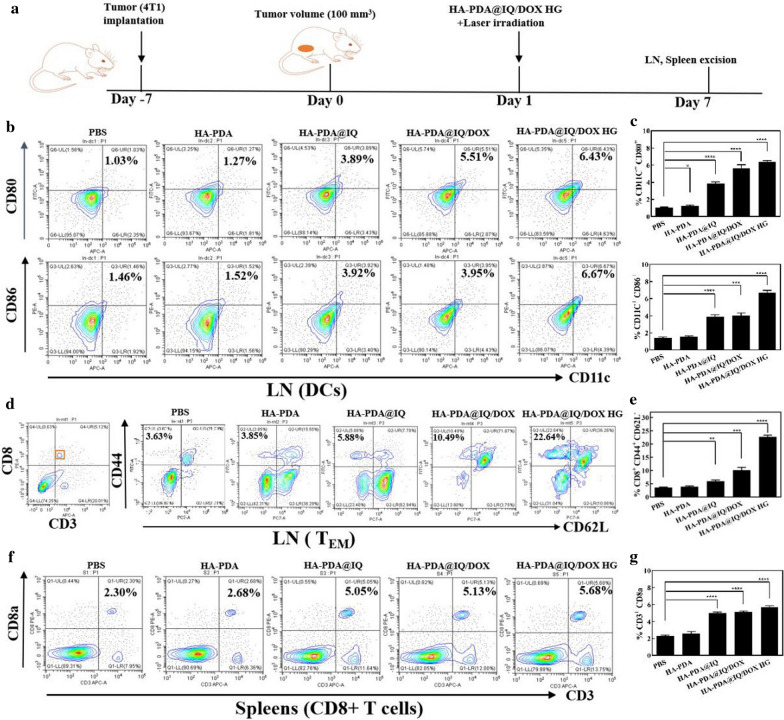
Immune responses assessment in vivo. **a** Scheme and timeline of the experimental design to evaluate the in vivo immune responses triggered by HA-PDA@IQ/DOX HG treatment. **b** Representative flow cytometry plots and statistic data **c** of CD80^+^ or CD86^+^ among CD11c^+^ DCs extracted from the inguinal lymph nodes (LNs). **d** Representative flow cytometry plots and statistic data **e** illustrated memory T cell in inguinal lymph nodes (LNs). **f** Representative flow cytometry plots and statistic data **g** of CD3^+^CD8a^+^T cells in spleen. *p* values were calculated by the Student’s t test. Mean ± SD (n = 4) of three independent experiments, *p < 0.05, **p < 0.01, ***p < 0.001, ****p < 0.001

Due to the Mn element doping, we speculated that HA-PDA@IQ/DOX NPs might possess the capability to be utilized as a potential contrast agent (CAs) for MR imaging. Herein, the 3 T MR scanner was used to study the *T*_1_-weighted MR of HA-PDA@IQ/DOX NPs in vitro. As shown in Fig. 6a, there was a positive correlation between HA-PDA@IQ/DOX NPs solutions with different Mn concentrations (0–0.2 nM) and *T*_1_-weighted MR signal intensity. Furthermore, HA-PDA@IQ/ DOX NPs encapsulated with F127 hydrogel was in situ injected into tumor site to access their imaging capability. As shown in Fig. 6b, strong signal could be observed in tumor region after HA-PDA@IQ/DOX NPs injection. Then, we further studied the retention and distribution of HA-PDA@IQ/DOX NPs at tumor site for 48 h. As shown in Fig. 7c , single HA-PDA@IQ/ DOX NPs disappeared quickly within 6 h, while HA-PDA@IQ/DOX HG still well remained in tumor region under 48 h. It could be seen that HA-PDA@IQ/DOX HG had a strong retention effect at tumor site, which was conducive to the recruitment of APCs as well as recognition of neoantigen derived from necrotic tumor cells.

**Fig. 8:**
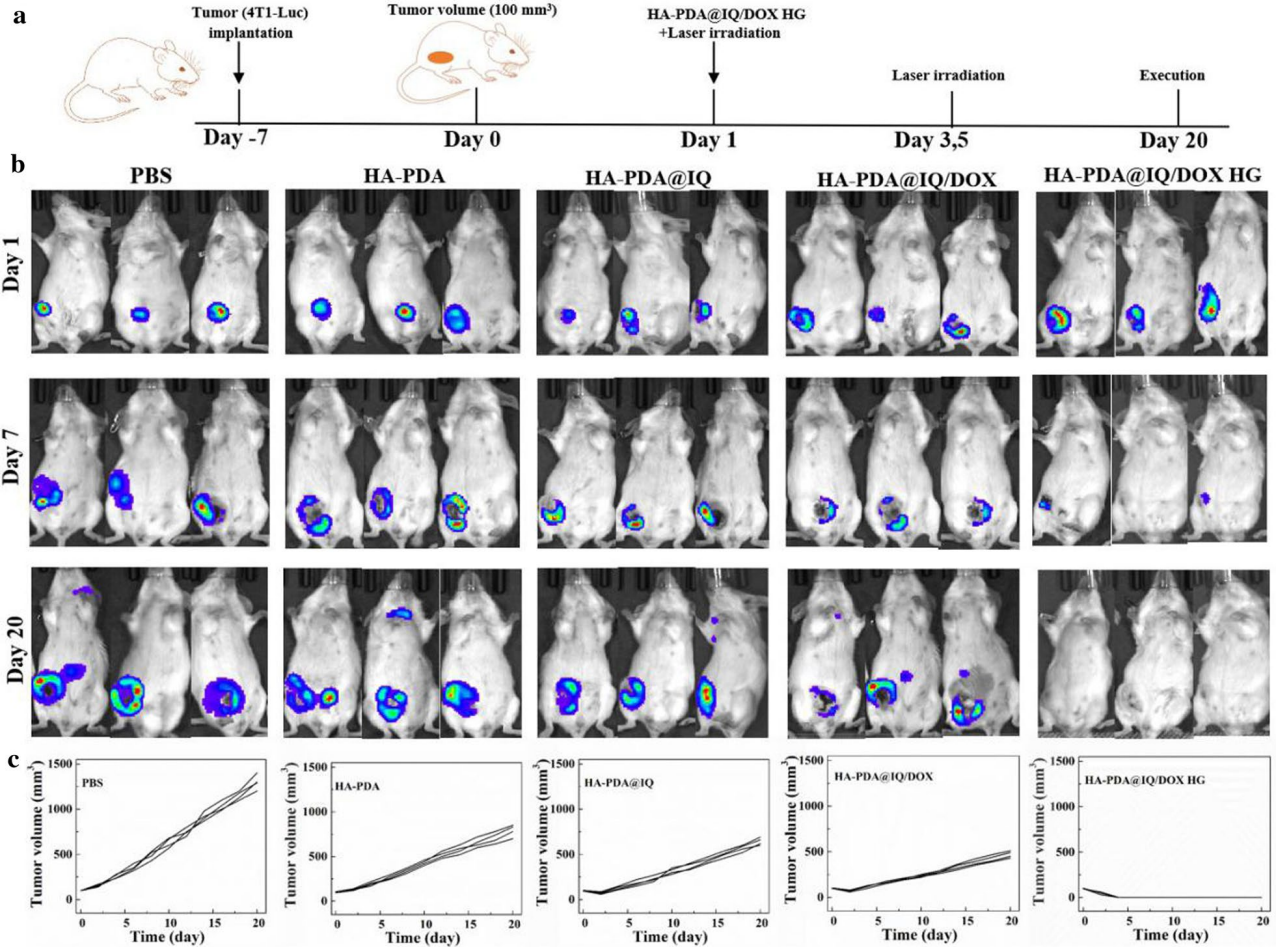
**a** Schematic diagram of experimental design for tumor therapy in vivo. **b** Luminescence imaging of 4T1-bearing mice and **c** corresponding individual tumor growth curves in different treatment until day 20 after treatment. (n = 4)

### Immune responses assessment in vivo

In view of the excellent photothermal performance and biocompatibility, we further studied the immunoregulatory function of HA-PDA@IQ/DOX NPs in vivo using 4T1 subcutaneous tumor-bearing mice as animal model (Fig. 7a). When the average tumor volume reached about 100 mm^3^, the 4T1 tumor-bearing mice were divided into five groups (PBS, HA-PDA NPs, HA-PDA@IQ NPs, HA-PDA@IQ/DOX NPs and HA-PDA@IQ/DOX HG) for in-situ injection under the 808 nm NIR irradiation (2 W/cm^2^, 5 min). Firstly, DOX was rapidly released from surface of HA-PDA@IQ/DOX NPs and killed tumor cells under the tumor microenvironment and hyperthermia. Afterward, the IQ was gradually released from HA-PDA@IQ/DOX NPs due to the instability of polydopamine nanoparticles under acidic environment, finally resulting in the activation of DCs via TLR7 signal pathway. The maturation of DC cells in the inguinal lymph nodes of mice with breast cancer was detected by flow cytometry (Fig. 7b and c). The data showed that the ratio of CD11c^+^/CD80^+^ (1.27%) and CD11c^+^/CD86^+^ cells (1.52%) in HA-PDA treatment group was a little bit higher than that in control group PBS (1.03%, 1.46%), which may be attributed to photothermal treatment influence. Notably, the proportion of CD11c^+^/CD80^+^ cells and the proportion of CD11c^+^/CD86^+^ cells in HA-PDA@IQ NPs treatment group were higher than those in HA-PDA NPs treatment group, confirming that immune adjuvant IQ doping could stimulate DC maturation. Meanwhile, the ratio of CD11c^+^/CD80^+^ and CD11c^+^/CD86^+^ cells in HA-PDA@IQ/DOX HG group were higher than that in HA-PDA@IQ/DOX NPs group. In the HA-PDA@IQ/DOX HG group, the DC maturation rate was the highest (6.43% for CD11c^+^/CD80^+^ cells and 6.67% for CD11c^+^/CD86^+^ cells). The reason may be that the addition of the gel prolonged the residence time of the nanoparticles in the tumor and enhanced the recruitment of APC cells. In order to study the potential utility of this treatment strategy for the long-term prevention of tumor recurrence, we used flow cytometry to detect the inducibility of effector T cells in inguinal lymph nodes (CD3^+^/CD8^+^/CD44^high^/CD62L^low^). As shown in Fig. 7d and e**,** the ratio of memory T cells reached 22.64% under HA-PDA@IQ/DOX HG treatment, which was 6.2 times compared with control group (PBS). These results demonstrated that HA-PDA@IQ/DOX HG had the ability to promote memory T cells production.

 Cytotoxic T lymphocytes (called CTL or CD8^+^ T cells) are essential for the anti-tumor immune response where they are activated by tumor-derived antigens and directly destroy target cells. In this experiment, each group of splenocytes was co-stained with anti-CD3 and anti-CD8a antibodies and was measured by flow cytometry. As shown in Fig. 7f and g, the percentage of CD3^+^/CD8^+^ T cells in PBS, HA-PDA NPs, HA-PDA@IQ NPs, HA-PDA@IQ/DOX NPs and HA-PDA@IQ/DOX HG group were 2.30%, 2.68%, 5.05%, 5.43% and 5.68%, respectively. It was worth noting that the percentage of CD3^+^/CD8^+^ T cells in HA-PDA@IQ NPs (5.05%) group was about 1.88 times as that in HA-PDA NPs (2.68%). This enhancement of CTL might be ascribed to the introduction of IQ, which remarkably stimulated the DC cells maturation and enhanced the tumor-derived antigens presentation function.

### The antitumor effect of HA-PDA@IQ/DOX HG in vivo

To verify the therapeutic effect of HA-PDA@IQ/DOX HG, subcutaneous 4T1-Luc breast tumor model was established in the inner mammary gland of lower left posterior breast (Fig. 8a). After 7 days of treatment, the tumors were remarkably inhibited in HA-PDA@IQ/DOX HG group compared with other groups in Fig. 8b**.** It was noted that the tumors in HA-PDA@IQ/DOX HG group completely disappeared on the 20th day. Individual tumor growth curves of each group further confirmed this trend (Fig. 8c). It can be seen that HA-PDA@IQ/DOX HG with irradiation possessed application potential in eliminating the tumor in situ.

**Fig. 9:**
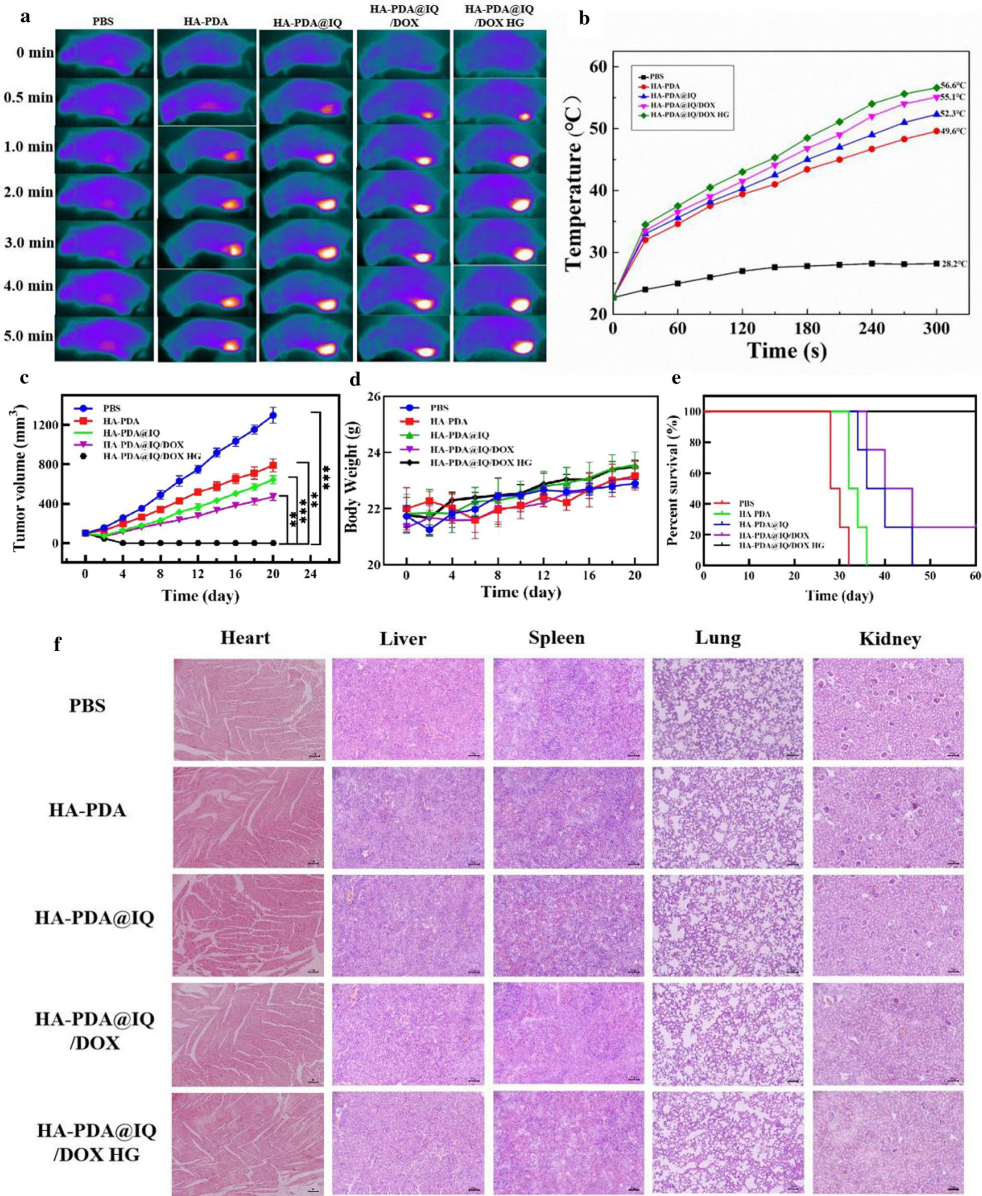
Characterization of HA-PDA@IQ/DOX HG mediated PTA using the 4T1 subcutaneous tumor-bearing mice model. **a** Infrared thermal images of mice at varied time points of treatment under different conditions using 808 nm laser irradiation. **b** Corresponding temperature change curves at the breast tumor sites of mice in different groups upon laser irradiation. **c** Tumor volume change curves, **d** the body-weight change, and **e** the survival curves of mice in the different groups. Data are expressed as mean ± S.D. (n = 4) (**P < 0.01, ***P < 0.001 by t-test). **f** H&E-stained images of the major organs in the different groups. Scale bar: 200 μm

### The PTA performance of HA-PDA@IQ/DOX HG in vivo

In order to evaluate the photothermal performance in vivo, digital NIR thermal imager was chosen to real-time monitor temperature profile of tumor site after in situ injection of HA-PDA@ IQ/DOX HG. As shown in Fig. 9a and b, the surface temperature of the tumor in each group increased sharply, except for the PBS group. Notably, the temperature of tumor in HA-PDA@IQ/DOX HG group reached its maximum and even up to 56.6 °C after 5 min of laser irradiation. After that, irradiation was performed every other day for a total of three times and corresponding tumor volume was recorded at certain time interval. As shown in Fig. 9c, the growth curves of the tumor within 20 days further verified that HA-PDA@IQ/DOX HG group exhibited optimal anti-tumor effect compared to other control groups. However, there was no significant change in body weight among all groups in Fig. 9d. It was worth noting that the survival period of the mice in HA-PDA@IQ/DOX HG group was as long as 60 days, while the average life span of mice in the control group was shorter than 30 days in Fig. 9e. The life span of mice in other treatment groups was extended to varying degrees, which was attributed to the lack of DOX and hydrogel collaboration. These results proved that PTA, hydrogel and chemotherapy could reinforce each other, finally obtain an ideal therapeutic effect. In addition, in order to verify its biological safety in vivo, the heart, liver, spleen, lung and kidney of the mice were taken out for H&E staining analysis (Fig. 9f). The images showed that there were no inflammatory lesions or obvious tissue damage.

## Conclusions

In this study, we reported that a novel NIR responsive nanovaccine (HA-PDA@IQ/DOX HG) for in situ PTA and chemotherapy can boost anti-tumor immunity. During the synthetic process, HA was used as template molecule to encapsulate IQ and assist dopamine oxidative polymerization catalyzed by potassium permanganate. In order to enhance the PTA-mediated ICD, the chemotherapeutic drug DOX was efficiently attached by HA-PDA@IQ NPs through π-π accumulation and electrostatic interaction. Upon NIR irradiation, the resultant HA-PDA@IQ/DOX NPs not only exhibited highly photothermal conversion efficiency of 41.2%, but also accelerated DOX release. In addition, HA-PDA@IQ/DOX NPs as CA were capable of MR imaging performance due to the introduction of Mn doping. Furthermore, HA-PDA@IQ/DOX NPs with favorable biocompatibility remarkably stimulated DC maturation in vitro. Most importantly, HA-PDA@IQ/DOX NPs loaded into thermosensitive hydrogel not only enable the repeated PTA, but also potentiated the antitumor immune responses. These results indicated that that HA-PDA@IQ/DOX HG possessed a promising potential to amplify antitumor immunotherapeutic efficacy.

## Experimental section

### Materials

Dopamine (DA), Hyaluronic acid (HA), Imiquimod (IQ) and Doxorubicin (DOX) were purchased from Aladdin Reagent Company (Shanghai, People's Republic of China). Cell counting KIT-8 (CCK-8) and 4', 6-diamidino-2-phenylindole (DAPI) were obtained from Thermo Fisher Scientific. Phosphate buffered saline (PBS) was purchased from Hyclone (Logan City, Utah, USA). Dulbecco’s Minimum Essential Medium (DMEM), RPMI Medium Modified (RPMI-1640) and Fetal bovine serum (FBS) were procured from Hyclone (Logan, UT, USA). Trypsin was obtained from Sigma-Aldrich Company (USA). Fluorescein diacetate (FDA) and Propidium iodide (PI) were purchased from Sigma (New York, NY, USA). De-ionized water was used in the experiments.

### Synthesis of HA-PDA@IQ/DOX NPs

0.1 g hyaluronic acid (HA) was dissolved in 200 mL deionized water, which was stirred continuously at room temperature until dissolved. Then, 0.1 g Dopamine (DA), 0.01 g Imiquimod (IQ) and 200 μL KMnO_4_ (10 mg/mL) were slowly added into the HA solution under continuous stirring for 2 h. The colorless solution immediately turned light yellow and gradually turned dark brown. Afterwards, the reaction solution was centrifuged at 3000 rpm for 10 min to remove the sediment. The supernatant was poured into a cut-off dialysis bag (MWCO 10,000) for dialysis for 5 days, changing the water every 6 h. The obtained dialysate was freeze-dried in a vacuum freeze dryer (Boyikang, Beijing, China) for 3 days to obtain a black powder (HA-PDA@IQ NPs). Finally, 1 mg/mL doxorubicin was added into 1 mg/mL HA-PDA@IQ NPs solution and stirred at room temperature for 24 h, then centrifuged at 10, 000 rpm for 20 min and collect the precipitate. The obtained precipitate was freeze-dried in a vacuum freeze dryer for 1 day to obtain HA-PDA@IQ/DOX NPs.

### Instruments and characterizations of HA-PDA@IQ NPs

The phase structure was analyzed by X-ray diffraction (XRD) on Rigaku smartlab 9kw (Rigaku, Japan), equipped with a Cu Kα radiation source (λ = 0.15418 nm), with a slit of 0.5° and a scanning speed of 7°/min. The X-ray photoelectron spectroscopy (XPS) was performed on Thermo Fisher Nexsa (America). UV–Vis absorption was identified by A560 dual-beam UV–Vis spectrophotometer (AOE Instruments, Shanghai). The morphology and the elemental mapping images of the HA-PDA@IQ/DOX were observed by transmission electron microscopy (TEM, FEI Talos F200S, America).

### Photothermal conversion efficiency

In order to measure the photothermal conversion efficiency (η), 200 μL of HA-PDA@IQ NPs aqueous solution (1 mg/mL) in the EP tube was irradiated using an 808 nm NIR laser with a power density of 2.0 W/cm^2^ for 600 s and then turn off the laser lasted 600 s. An infrared camera was used to monitor the temperature change of the solution for 1200 s. The η value of the sample was determined by the following formula[41, 42]:

$$\eta = \frac{{{\text{hS}}(\Delta T_{\max ,mix} - \Delta T_{{\max ,H_{2} O}} )}}{{I(1 - 10^{ - A808} )}}$$

$${\text{hS}} = - \frac{{\sum\limits_{i} {m_{i} C_{p,i} } }}{{\text{t}}}\ln \theta$$

$$\theta = \frac{\Delta T}{{\Delta T_{{{\text{max}},mix}} }}$$

In the equations, h is the heat transfer coefficient; S is the surface area of the container; *ΔT*_max,mix_ is the temperature change of the HA-PDA@IQ/DOX NPs dispersion at the maximum steady-state temperature.*ΔT*_max,H2O_ is the temperature change of water at the maximum steady-state temperature. *I* is the laser power, *A*_*808*_ is the absorbance of HA-PDA@IQ/DOX NPs at the wavelength of 808 nm in aqueous solution. Compared with solvent (water, 2 × 10^–3^ kg), mass of NPs (2 × 10^–8^ kg) was too little. Generally, the specific heat of water is much higher than other materials. Consequently, the m_NPs_ and *C*_p,NPs_ of NPs were neglected. *m*_H2O_ was 2 × 10^−3^ kg. Cp_,H2O_ was 4.2 × 10^3^ J/(kg·℃). *θ* is the dimensionless driving force temperature,and defined as the ratio of *ΔT* to *ΔT*_max,mix._

### Dox loading and release test

In order to load the DOX, HA-PDA@IQ NPs (1 mg/mL) were dissolved in PBS (20 mM) at pH 7.4. Then, DOX at distinct different weight ratio with HA-PDA@IQ (0.1, 0.2, 0.5,1, 2, 3, 3.5) were added and stirred for 24 h in the dark. The above solutions were centrifuged and washed with PBS solution until the supernatants were colorless. The loading capacity of DOX was detected by UV–Vis-NIR spectrum.

Then, the release of DOX from HA-PDA@IQ/DOX was studied under pH 5.0 and 7.4 at different times. The amount of DOX release was also measured by UV–Vis-NIR spectroscopy. In addition, the release of DOX was simulated by NIR under 808 nm laser irradiation. In the experiment, HA-PDA@IQ/DOX was dissolved in 15 mL PBS with different pH values (5.0 and 7.4). At different time points, 808 nm laser was used to irradiate with a power density of 2 W/cm^2^ for 5 min. Afterwards, 1 mL of the solution was collected and centrifuged to obtain the released DOX, which was measured and determined by UV–Vis spectroscopy. Three replicates were carried out.

### Biocompatibility of HA-PDA@IQ NPs

The CCK-8 assay was used to evaluate the biocompatibility of HA-PDA@IQ NPs by incubating with 4T1 cells (mouse breast cancer cell line) and MEF cells (mouse embryo fibroblast cell line) respectively. Both of cells were inoculated in a 96-well plate with approximately 1 × 10^4^ cells in each well at 37 °C and 5% CO_2_. When the cell density reached 60%, the medium was discarded and replaced with new medium containing different concentrations of PDA@IQ NPs (0, 200, 400, 600, 800, 1000, 1200 μg/mL). After incubation for 48 h, these cells were washed with PBS and incubated with 10 μL CCK-8 and 90 μL DMEM or RPMI-1640. After 2 h, the absorbance value of the solution at 450 nm was measured by ultraviolet spectrophotometer (BioTek Instruments, Inc Vermont, USA). The experiment was repeated with three parallel groups. The relative cell viability was calculated as (Abs _sample_ − Abs _zero sitting_)/(Abs _control_ − Abs _zero sitting_) × 100%.

### Hemolysis test

First, 100–150 μL of 10% chloral hydrate solution was administrated into the mouse by intraperitoneal injection. After the deeply anesthetizing, the mouse’s eyeballs were removed with tweezers. The fresh eye blood of the mouse was quickly collected into a centrifuge tube with heparin-infiltrated tube wall and centrifuged for 15 min at a speed of 1200 rpm. The supernatant was discarded and an appropriate amount of PBS was added into the centrifuge tube to wash twice until solution was clear. Finally, the below red precipitate was mouse red blood cells (MRBCs). 100 μL of the diluted MRBCs was added into the EP tube, mixed with 900 μL of HA-PDA@ IQ/DOX with different concentrations (100,200, 400, 800, 1000, 1200 μg/mL), PBS as negative control, de-ionized water as positive control. All samples were allowed to stand at room temperature for 2 h, then centrifuged at 1000 rpm for 1 min. The supernatant was carefully transferred to the new EP tube and measured the absorbance using a dual-beam UV–Vis spectrophotometer at 541 nm.

### Phagocytosis experiment

Confocal laser scanning was performed to study the phagocytosis and distribution of HA-PDA@IQ/DOX in cells. A small disc was pre-installed at the bottom of the 24-well plate. Then, 4T1 cells were seeded into the 24-well plate at 1 × 10^4^ cells per well in an incubator at 37 °C and 5% CO_2_ for 24 h. When the cell density reached 60%, the medium was replaced with new medium containing 50 μg/mL HA-PDA@IQ/DOX NPs under the same conditions for 6 h. Next, the medium containing HA-PDA@IQ/DOX nanoparticles was discarded and washed twice with PBS. 0.5 mL of 4% paraformaldehyde was added into each well to fix for 15 min at room temperature. After washed 3 times with PBS, 300 μL DAPI working solution was added to each well and stained for 5 min at room temperature in the dark. The cells were washed 3 times with PBS for 10 min each. Finally, the small discs were taken out and placed upside down on a glass slide pre-dropped with appropriate glycerin, sealed with a neutral resin and stored at 4 °C in the dark. These samples were observed under confocal laser scanning microscopy.

### DC maturation in vitro

Bone marrow-derived dendritic cells (BMDCs) were obtained from bone marrow precursors of 6-week-old Balb/c female mice [40]. Then, BMDCs were seed in a 6-well plate (1 × 10^6^ cells/well). For in vitro stimulation experiments, BMDC was co-cultured with HA-PDA@IQ NPs at different concentrations (0, 10, 100, 200, 400, 800 μg/mL) for 48 h. At the optimized conditions, the loading capacity of IQ in is 1.2%, the dosages used to induce DC maturation in vitro are 0, 0.12, 1.2, 2.4, 4.8, 9.6 μg/mL (2 mL), respectively. DCs were collected and stained with anti-CD11c FITC, anti-CD86 PE and anti-CD80 APC for flow cytometry analysis.

### Mouse tumor model

In our experiment, female Balb/c mice were purchased from Jiangsu ALF Biotechnology Co., LTD. Animal experiments were carried out in accordance with the protocol approved by the Experimental Animal Center of Jiangsu University. To establish a tumor model, 4T1 cells (1 × 10^6^) in 20 μL PBS were subcutaneously injected into the lower left breast of each mouse. For in vivo combination therapy, 4T1 tumor-bearing mice were divided into five groups, including PBS, HA-PDA NPs, HA-PDA@IQ NPs, HA-PDA@IQ/DOX NPs and HA-PDA@IQ/DOX HG. After around one week, the average size of the tumor reached about 100 mm^3^, and then about 1 mg/mouse of each group of nanoparticles was injected in situ. The dose of IQ is about 12 μg/mouse and the dose of DOX is about 180 μg/mouse and the concentration of thermosensitive hydrogel is 20%. The tumor was irradiated with 808 nm laser at a power density of 2 W/cm^2^ for 5 min. During the laser irradiation, the temperature change of the tumor was recorded by an infrared thermal imaging camera (HT-19). After treatment, Tumor size was monitored by vernier caliper to record the lengths and widths every two days for two weeks. The tumor volumes were calculated by “length × width^2^/2”.

### Measurement of Memory T Cell, DC cell and CD8^+^ T cell populations.

Seven days after challenging mice with 4T1 tumor, the inguinal lymph nodes and spleen were isolated and dissociated into single cells by mashing through cell strainers (70 μm). The cell suspension of inguinal lymph nodes was stained with APC anti-mouse CD3 (BioLegend), PE anti-mouse CD8a (BioLegend), PE/Cy7 anti-mouse CD62L (BioLegend), FITC anti-mouse/ human CD44 (BioLegend) and APC anti-mouse CD11c, FITC anti-mouse CD80, PE anti-mouse CD86 and APC anti-mouse CD3, PE anti-mouse CD8a, respectively. The cell suspension of spleen was stained with APC anti-mouse CD3 (BioLegend), PE anti-mouse CD8a (BioLegend). The percentage of CD3^+^CD8^+^CD44^high^CD62^low^ cells, CD11c^+^CD80^+^CD86^+^ cells, and CD3^+^CD8^+^ corresponding to effector memory T cells, DC cells, CD8^+^ T cells was analyzed, respectively.

### MR imaging

The Skyra 3.0 T superconducting magnetic resonance imaging system (MAGNETOM Trio TimSystem, Siemens, Germany) was used to scan different concentrations of HA-PDA@IQ/DOX NPs aqueous solution (0–0.20 nM Mn). *T*_1_-weighted animal MR imaging was performed on the same MR scanner with a special coil specially designed for small animal images, the images were obtained under the following parameters: TR/TE = 600/9.2 ms, slices thickness = 2 mm, 256 × 256 matrices.

### Histological analysis

In order to conduct biocompatibility studies, five groups of mice with different treatments were sacrificed and the major organs (heart, spleen, liver, lung, kidney) were collected. These organs were immersed in 4% paraformaldehyde buffer solution for 16 h, then trimmed, dehydrated, transparent treated and embedded in paraffin wax. The samples were sliced serially at a thickness of 5 μm and stained with hematoxylin and eosin. Finally, observe the tissue section under an optical microscope.

### Statistical analysis

All experiments were repeated at least three times, and the results were expressed as mean ± SD. The statistical significance of all results was determined by the student's* t* test. *P* < 0.05 was considered statistically significant.

## Data Availability

The data sets supporting the results of this article are included within the article.
